# Parental practices and perspectives on unused pediatric medications at home: A community-based cross-sectional study in Burdur-Türkiye

**DOI:** 10.1371/journal.pone.0344779

**Published:** 2026-03-13

**Authors:** Serkan Köksoy, Belkıs Can

**Affiliations:** 1 Burdur Mehmet Akif Ersoy University, Health Science Faculty, Burdur, Türkiye; 2 Aydın Adnan Menderes University, Vocational School of Health Services, Aydın, Türkiye; Kampala International University - Western Campus, UGANDA

## Abstract

**Background:**

Recently, many scientific studies have examined the handling and disposal practices of unused medicines in adults, but research focusing on parental management of pediatric pharmaceuticals remains limited. Therefore, this study aims to determine the practices and perspectives of parents about unused pediatric pharmaceuticals, to measure the amount of unused pediatric pharmaceuticals and to classify medicines in Burdur-Türkiye.

**Methods:**

This study was designed as a cross-sectional study and conducted in Burdur-Türkiye. Data were collected between January 1, 2024, and January 31, 2025, using convenience sampling, with each participant visited in their household. Pharmaceuticals were classified according to both mechanism of action and ATC. Data were analyzed with SPSS v27.

**Results:**

The study included 227 participants and 558 unused pediatric pharmaceuticals were obtained from them. It was found that NSAIDs (21.3%), analgesics and antipyretics (14.5%) and antibiotics (12%) were the most commonly kept at home as unused medicines. It was found that the reason for keeping leftover medicines was early recovery (51.2%), that expired medicines were thrown in the household garbage (85.5%), and that the mother was the one who most often gave the medicine to the child (84.1%). In the participants’ opinion, when only the “yes” option was considered, the prevalence of self-medication was 45.8%. When the “yes and sometimes” options were combined, it was 82.4%, and the frequency was high for antipyretics, analgesics, and cold medicine, but low for antibiotics (1.3%). In participant practice, the prevalence was 58.4%, and 40.3% for antibiotics. A statistical difference was found between participants’ opinions and practices (p < 0.001), and Cohen’s k was −0.036 (95% CI: −0.215–0.137). For antibiotics, it was −0.212 (95% CI: −0.556–0.222) and p < 0.001. The volume of unused medication Median(25–75;IQR) was 56(27–80;53) ml and its percentage was 51.7% for all medications, and 43(22–69;47) ml and 47% for antibiotics (respectively). According to ATC, drugs coded *R05X-Other Cold Preparations* (n = 85 and 15.2%), *N02BE01-Paracetamol* (n = 77 and 13.8%) and *M01AE01-Ibuprofen* (n = 73 and 13.1%) were the most commonly unused pediatric drugs.

**Conclusion:**

There are significant amounts of unused medicines at homes and the amounts of used and unused medicines are close to each other. Self-medication is a common behavior in opinion and practice among parents, and the findings regarding antibiotics are remarkable. Reported behaviors and viewpoints concerning unused medications among adults show parallels with the findings of this study regarding pediatric pharmaceuticals, which is a concerning situation. Since medication storage areas are largely accessible to children, precautions should be taken against potential risks. Information on unused pediatric pharmaceuticals should be added to trainings on drug use. Individuals, expectant mothers, pregnant women and mothers should be informed about pediatric pharmaceuticals. An economical and efficient drug take-back system should be established.

## Introduction

The management, handling, and disposal of unused medicines is a topic that has been frequently addressed recently, and the findings emphasize that it is an important public health issue that could pose serious risks to our future if not addressed. There are a large number of epidemiologic studies on this topic and the most recent ones have been reported from developing countries such as Ethiopia, Pakistan, Liberia, Nigeria, Palestine and Saudi Arabia [1–6]. Among these studies, there are some findings that have come to the forefront. These findings include a significant number of unused and expired medicines, lack of knowledge on how to dispose of them, incorrect disposal methods, lack of laws and regulations to control the situation, and self-medication. These reports show us that the problem is, in fact, a major public health problem that is widespread throughout the world. At the same time, there are studies in developed countries (USA, Italy, Japan) that report that these problems have not been fully resolved and are still under research [7]. The above-mentioned situation may be related to many factors. One of them is shown as lack of knowledge/education, but this factor may not be as reported. Because it is observed that the findings obtained from the studies in which students who are participants receiving education and training on medicines are close to the medication-related behaviors and viewpoints of the general public [8–11]. Failure to handle medicines responsibly can contaminate vital structures such as soil and water, pose a danger to living organisms and pose a threat to the future of the world [12,13]. Various researches are being conducted in some countries to solve this problem. These researches are mainly focused on drug take-back systems [14].

In addition to studies on the general public, various studies have been conducted on leftover or unused pediatric pharmaceuticals. One of the most important issues addressed in pediatric pharmaceuticals is self-medication, which is the fact that a parent gives a medicine to his/her child without consulting the physician. Numerous studies have been published addressing this issue and researchers have generally focused on antibiotics [15–17]. The findings of these studies included the reasons for self-medication. If we list these reasons; they are the fact that parents have to give medication to their children due to the distance to health facilities, their past experiences and the fact that they find it easier and more feasible to give the medicines at home to their children for similar situations experienced in the past [18,19].

As can be seen from the current literature, the issue is widely addressed both in the general public and pediatrics, and continues to be predominantly focused on antibiotics in pediatrics. However, studies addressing the situation regarding pediatric pharmaceuticals in Türkiye have been extremely limited. Therefore, this study aims to determine the practices and perspectives of parents about unused pediatric pharmaceuticals, to measure the amount of unused pediatric pharmaceuticals and to classify medicines in Burdur-Türkiye.

## Materials and methods

### Study design

This study was designed as a cross-sectional study and conducted in Burdur-Türkiye. Data were collected between January 1, 2024, and January 31, 2025, using convenience sampling, with each participant visited in their household. The researchers conducted face-to-face meetings with parents in locations such as elementary schools and family health centers to reach potential participants. The purpose and procedures of the study were explained to parents, and appointments were scheduled with those who were eligible and consented to participate in the study.

### Criteria for participants and medicines

In this study, detailed participant and medication inclusion-exclusion criteria were applied to clearly delineate the scope of parental practices and experiences concerning unused pediatric medications at home.

### Participant criteria

Those who accepted the preliminary information, gave consent, answered the data form completely, and parents with unused medicines in their possession were included and the following criteria were detailed. In Türkiye, the form of the medicine can be determined based on the physical and developmental characteristics of a child (syrup instead of capsule, etc.). Children under 12 years of age are usually prescribed medicines in the form of syrups, solutions and suspensions and it is recommended that these medicines are administered by parents. Since pediatric pharmaceuticals will be measured volumetrically in this study, another criterion was determined as “having a child under 12 years of age”. In the preliminary study (n = 12), “having one child” was determined as another criterion since it was observed that parents confused the medications when there was more than one child or when their ages were close to each other (the findings of 12 participants were not included in the findings of the study). Another inclusion criterion for questions related to giving medication to the child was that parents live together as a family (one parent from each home who is most involved in administering the child’s medication). Another inclusion criterion is that the child does not have a known illness and does not use the medicines at home for any illness. Participants who did not meet the above criteria were excluded from this study.

### Drugs criteria

A detailed inclusion criteria was applied for unused pediatric liquid form drugs. These medications which parents had obtained for their children either by physician prescription or over-the-counter, had been previously used but were currently not being administered, were not planned for use after measurements, and had expired were included in the study. Unused drugs with a Defined Daily Dose (D.D.D)<1, i.e., those with less than one dose left, were considered finished and excluded from this study. Medicines for which parents did not give permission for measurement (drugs in liquid form that were leftover but could be used in the future and whose expiry date had not expired) were considered as “drugs under treatment”, summarized in number in the relevant table and volumetric measurements were not made from these drugs.

### Unused pharmaceutical

These are pharmaceuticals taken by parents for the treatment of the child, administered to the child, leftover after treatment and not planned for use. The forms of these medicines for children include syrup, suspension and solution. The unused medications were checked and approved by both the participants and the authors according to the relevant criteria before being included in the study. The unused medicines were withdrawn from the medicine packaging with 1 ml, 2.5 ml, 5 ml, 10 ml syringes and the amount of unused medicine was determined. For example: If 30 ml was obtained from “*Paracetamol 100 ml Suspension*” by syringe, it was evaluated as “*used=70 ml (70%), unused=30 ml (30%) and total 100 ml (100%)*”.

### Classification of drugs

Two classifications were made for unused drugs. The first classification was based on the mechanism of action of the drug (Antibiotics, NSAIDs, Analgesics etc.) and was used in some previous studies [20]. The other classification was based on the Anatomical, Therapeutic, Chemical (ATC) index and was used in previous studies by the authors [11, 21]. The ATC index is an important source of classification presented by the World Health Organization (WHO) and WHO Collaborating Centre for Drug Statistics Methodology [22].

### Sample size

Sample size was calculated with the G*power program [23]. In this program, the input data are entered as t tests-Means: Wilcoxon-Mann-Whitney test (two groups), options = ARE method, Parent distribution = Normal, Effect size d = 0.5, α err prob = 0.05, Power (1-β err prob) =0.95, Allocation ratio n2/n1 = 1. Output data were calculated as Non centrality parameter δ = 3.6, Critical t = 1.97, Df = 208,1, Group 1 = 110, Group 2 = 110, Total sample size = 220 and Actual power = 0.95.

### Data collection tool

A structured data collection form was developed based on a review of current literature [2,21,20,24,25]. The question items were drafted by the authors, translated and back-translated by linguistic experts, and their clarity, relevance, and comprehensiveness for the study’s objectives were evaluated by five experts in epidemiological research (Kendall’s W coefficient of concordance = 0.71). A pilot study was conducted with 12 participants to assess the comprehensibility, feasibility, and approximate completion time of the form. Based on the feedback from the pilot study, minor wording adjustments were made to improve clarity. Data from the pilot study were not included in the final analysis. The first part of the form collected information on the sociodemographic characteristics of the parents and children, along with items exploring parental medication use and storage practices. The second part contained questions regarding the characteristics of the unused medications and a section to record their quantity.

### Data analysis

SPSS v27 package program was used for data analysis. Data related to descriptive parameters are given as n, %, mean (X), standard deviation (S), median, percentile 25, percentile 75 and inter quantile range (25–75;IQR). Sociodemographic parameters and the number of medications used and unused were analyzed with Mann-Whitney U and Kruskall-Wallis. Comparison of the amounts of used and unused drugs was performed with Mann-Whitney U test. The difference between opinions and practices was analyzed using Cohen’s k. Statistical significance value was taken as p < 0.05.

### Ethics committee

This study was approved by Burdur Mehmet Akif Ersoy University Non-Interventional Clinical Research Ethics Committee (No:2023/553). All participants provided written informed consent prior to their involvement. The consent form detailed the study’s purpose, procedures, potential risks and benefits, confidentiality safeguards, and the voluntary nature of participation, including the right to withdraw at any time without penalty.The study was conducted in accordance with Declaration of Helsinki.

## Results

A total of 236 (Response rate 16%) people were interviewed for the study, one parent from each home, and the data of 9 participants were excluded due to various reasons (missing data, privacy, outliers, etc.). As a result, 227 participants were included in the study and 558 medicines were obtained from these participants.

The mean age of the participants was 35.7 ± 6.6 years (range:18–56). The age of their children was 8.2 ± 2.9 years (range:3–12), height was 127.5 ± 22.3 cm (range: 70–165) and weight was 32.3 ± 13.4 kg (range:9–66). Analysis of the sociodemographic characteristics of the participants is summarized in Table 1.

**Table 1:** Comparison of sociodemographic characteristics with drugs (n = 227).

Variable		n(%)	Number of drugs under treatment			Number of unused drugs		
			Median (25^th^-75^th^)	Test	p	Median (25^th^-75^th^)	Test	p
Gender	Female	202 (89)	1 (0–2)	U:2.503	0.94	2 (1–3)	U:2424.5	0.74
	Male	25 (11)	1 (0–2)			2 (2–3)		
Place	District	52 (22.9)	2 (0–3)	U:3784	**0.049**	3 (2–4)	U:3549	**0.013**
	City	175 (77.1)	0 (0–2)			2 (1–3)		
Economic	I = E	176 (77.5)	1 (0–2)	KW:0.23	0.89	2 (1–3)	KW:0.38	0.83
	I < E	22 (9.7)	1 (0–3)			2 (2–3)		
	I > E	29 (12.8)	1 (0–2)			2 (1–3)		
Education	Primary	43 (18.9)	2 (0–3)^a^			2 (1–3)^a^		
	Secondary	88 (38.8)	1 (0–2)^ab^	KW:12.3	**0.002**	3 (2–4)^a^	KW:6.47	**0.04**
	Higher	96 (42.3)	0 (0–2)^b^			2 (1–3)^a^		
Employment	Working	105 (46.3)	1 (0–2)	U:6279	0.79	2 (1–3)	U:5115	**0.007**
	Not working	122 (53.7)	1 (0–2)			3 (2–3)		
Child	Girl	114 (50.2)	0 (0–2)	U:6354	0.85	2 (1–3)	U:6074.5	0.45
	Boy	113 (49.8)	1 (0–2)			2 (2–3)		

n: participant, %: Percentage, I: Income, E: Expense, Quartile: 25^th^-75^th^, U: Mann-Whitney-U, KW: Kruskal-Wallis, Post-hoc: Tamhane and indicated by a letter.

Percentages of female, city, income-expense balanced, higher education, non-working and having a daughter were higher than the other options. The mean number of medications under treatment was higher for male, district, low income, primary education, non-working and having a daughter. Statistical difference was found only in the parameters of place and education (between primary and higher education) (p < 0.05). In the mean number of unused drugs, the mean of male, district, low income, secondary education, non-working and having a male child were higher. Statistical difference was found in the parameters of place, education and employment (p < 0.05). In all participants, the number of drugs under treatment was 1.18 ± 1.43 (Max: 6 and total: 267), the number of unused drugs was 2.45 ± 1.28 (Max: 7 and total: 558) and the number of all pediatric pharmaceuticals at home was 3.63 ± 1.91 (Max: 11 and total: 825) (Table 1).

The percentage of kitchen as the storage room and refrigerator as the storage area, Family physician as the branch that prescribes medication the most, the possibility of reuse in emergencies, checking the expiry date, reading the prospectus and checking the medication before administering medication to the child as the reason for keeping medication at home were higher than the other options. The prevalence of self-medication was 45.8% when only the “yes” option was considered. When the “yes and sometimes” options were combined, it was observed to be 82.4% and it was high with antipyretics, analgesics, and cold medicines, and low with antibiotics (1.3%). These self-medication findings were evaluated as “participant opinion”. The frequency of buying drugs (without consulting a physician and without a prescription) was 33% and the most important reason for leftover drugs was related to early/quick recovery. The percentages of the options of keeping unused medicines at home until the expiry date, throwing away expired medicines and administering medicines to children mostly by the mother were found to be higher than the other options (**Table 2**).

**Table 2:** Parental practices and perspectives on unused pediatric medications.

Questions	Options	n	%
Room where the drug is stored*	Kitchen	204	85.4
	All other areas	35	14,6
Drug storage space*	Refrigerator	170	68.3
	Kitchen cabinet	64	25.7
	Wardrobe	15	6
The department that most often prescribes drugs for your child*	Family physician	120	45.8
	Emergency physician	34	13
	Pediatrician	108	41.2
Reason for keeping unused drugs at home*	To use it again	163	44.7
	For emergencies	164	44.9
	To give to someone else	31	8.5
	Other	7	1.9
Checking the expiration date	Yes	218	96.1
	Sometimes	8	3.5
	No.	1	0.4
Prospectus reading	Yes	182	80.2
	Sometimes	37	16.3
	No.	8	3.5
Checking before giving the drug	Yes	178	78.4
	Sometimes	37	16.3
	No.	12	5.3
Administering drug without consulting a physician/pharmacian	Yes	104	45.8
	Sometimes	83	36.6
	No.	40	17.6
If your answer to the previous question is “yes or sometimes”; drugs administered without consulting a physician (self-medication “opinion”) *	Antibiotics	11	1.3
	Antipyretics	156	18.4
	Cough drugs	120	14.2
	Common Cold Drugs	135	15.9
	Painkillers	147	17.3
	Vitamins	86	10.1
	Creams-Pomades etc.	106	12.5
	Nasal Sprays and others	87	10.3
Drug purchasing behavior (without consulting a physician and without prescription)	Yes	75	33
	No.	152	67
Reason for drugs being left over*	Early recovery	205	51.2
	Diagnosis-treatment change	82	20.4
	Side effect	55	13.7
	Not useful	22	5.5
	The child does not want to	22	5.5
	Other	15	3.7
Consequence of unused medicine*	Retained until expiry	176	52.5
	Thrown in the trash	91	27.2
	Given to the health facilities	28	8.4
	Given to the pharmacy	22	6.5
	Given to those in need	14	4.2
	Other	4	1.2
Disposal of expired drugs*	Thrown in the trash	213	85.5
	Other	36	14,5
The parent that most often administers drugs tothe child*	Mother	191	84.1
	Father	36	15.9

* Multiple options can be marked. n = Number of participant opinions, %: Percentage of participant opinion.

### Evaluation of drugs

In the study, 558 medicines were evaluated. It was observed that these medicines, all of which were in liquid form, were in syrup (n = 321, 57.5%), suspension (n = 173, 31%) and solution (n = 64, 11.5%) formats and their volumes ranged between 5 ml and 250 ml. When this finding was detailed, it was found that there were 250 ml (1), 200 ml (27), 150 ml (121), 125 ml (15), 120 ml (27), 100 ml (293), 80 ml (1), 75 ml (11), 70 ml (11), 60 ml (5), 48 ml (3), 40 ml (1), 30 ml (9), 20 ml (2), 18 ml (5), 15 ml (2), 10 ml (22), 5 ml (2) (drug quantities are given in parentheses). Of the 558 drugs analyzed, 484 (86.7%) were prescription, 54 (9.7%) were non-prescription/purchased and 20 (3.6%) could not remember prescription/non-prescription.

For each drug, the participant was asked about self-medication (*Did you give this drug to your child without consulting a physician/pharmacian?*) and the prevalence of self-medication was 58.4% (n = 326) for 558 drugs. When these numbers were detailed by mechanism of action, 71 of 119 NSAIDs (59.7%), 54 of 81 analgesics and antipyretics (66.6%), 35 of 64 antihistamines (54.6%), 32 of 56 non-opioid antitussives (57.1%), 23 of 36 cold medicines (63.9%), 24 of 31 decongestants (77%), 4%), 16 of 28 vitamins and minerals (57.1%), 12 of 18 antiseptics and local anesthetics (66.6%), 9 of 18 prokinetics (50%), 8 of 15 mucolytics (53.3%), 6 of 12 bronchodilators (50%), 9 of 13 others (69.2%) and 27 of 67 antibiotics (40.3%) were self-medicated. In the top three according to ATC classification, it was found to be 50 (71.4%) of 70 N02BE01-paracetamol, 56 (65.1%) of 86 R05X-Other cold preparation, and 40 (54.8%) of 73 M01AE01-Ibuprofen. These self-medication prevalence’s were considered *“participant practice”.* Cohen’s k was calculated as −0.036 (95% CI: −0.215–0.137) for participant opinion and participant practice in all medications. For antibiotics, it was −0.212 (95% CI: −0.556–0.222).

### Evaluating unused drugs according to their mechanism of action

When the drugs were classified according to their mechanism of action, 13 classes were observed. The evaluation and analysis of the used and unused amounts of the drugs in ml are given in Table 3.

**Table 3:** Classification of unused drugs (ml) according to their mechanism of action.

Action of Medicines	n	%	Drug	Median(25–75;IQR)	Test(U)	p
Analgesic and Antipyretic	81	14.5	Used	66 (34–98;64)	2709.500	0.056
			Unused	79 (48–112;64)		
Antibiotics	67	12	Used	45 (30–66;36)	2035.500	0.352
			Unused	43 (22–69;47)		
Antihistaminics	64	11.5	Used	43 (20.5–66;45,5)	1249.500	**<0.001**
			Unused	68 (43–111;68)		
Antiseptics and Local anesthetics	18	3.2	Used	35.5 (13–65;52)	96.500	**0.038**
			Unused	71 (19–146;127)		
Bronchodilators	12	2.2	Used	74.5 (31–112;81)	65.000	0.686
			Unused	57 (38–91;53)		
Decongestants	31	5.6	Used	8 (6–12;6)	272.500	**0.003**
			Unused	4 (2–8;6)		
Mucolytics	15	2.7	Used	41 (23–74;51)	84.000	0.237
			Unused	61 (28–83;55)		
Non-opioid Antitussive	56	10	Used	51 (32.5–80.5;48)	1538.000	0.861
			Unused	54 (32–82.5;50.5)		
NSAIDs	119	21.3	Used	47 (29–71;42)	6671.000	0.441
			Unused	53 (29–71;42)		
Prokinetics	18	3.2	Used	60.5 (46–105;59)	156.500	0.862
			Unused	77 (47–85;38)		
Common Cold	36	6.5	Used	42.5 (31–71;40)	578.500	0.434
			Unused	57.5 (29–69;40)		
Vitamins and Minerals	28	5	Used	49 (35.5–66;30.5)	370.500	0.725
			Unused	51 (34–63.5;29.5)		
Others	13	2.3	Used	40 (21–58;37)	82.000	0.898
			Unused	33 (19–86;67)		
**Total**	**558**	**100**	Used	46 (25–73;48)	140424.500	**0.005**
			Unused	56 (27–80;53)		

Median(25–75;IQR) (ml), Median(Percentile 25–75;Inter Quantile Range), Test (U): Mann-Whitney U, Medicines with less than 5 drugs in a row are given as “others”. Others (n = 13): Antiacids = 2, Antifungal = 3, Antispasmodics = 4, Antiviral = 4.

There was no statistical difference (p > 0.05) between the amount of used and unused drugs in the class of analgesics and antipyretics, antibiotics, bronchodilators, mucolytics, non-opioid antitussives, NSAIDs, prokinetics, cold medicines, vitamins-minerals and other, meaning that the amount of used and unused drugs were close to each other. Statistical difference was found only in antihistamines, antiseptic-local anesthetics, decongestants and total (p < 0.05). The highest volume of unused drugs was in antiseptic-local anesthetics, while the highest volume of used drugs was in bronchodilators (Table 3).

In the grouping according to mechanism of action, the volumes of unused drugs were analyzed as percentages (not given in Table 3). Accordingly, analgesics and antipyretics were 53.3%, antibiotics 47%, antihistamines 59.5%, antiseptics-local anesthetics 63.3%, bronchodilators 48.9%, decongestants 39%, mucolytics 55.7%, non-opioid antitussives 50.2%, NSAIDs 51.3%, prokinetics 49.9%, cold drugs 52%, vitamins-minerals 50.5% and others 53.1%. The volumetric percentage of unused medication was 51.7% for all drugs.

### Evaluating unused drugs according to ATC

In the classification according to ATC, it was observed that there were 8 classes and the highest diversity was observed in the R-Respiratory System class. The percentage classification according to ATC codes is summarized in Table 4.

**Table 4:** Percentage of used-unused medicines according to ATC codes.

Class	ATC Code	ATC Name	n	%	%Used	%Unused
A-Alimentary tract and metabolism	A12CB01	Zinc Sulfate	19	25	49.9	50.1
	A03FA01	Metoclopramide	14	18.4	53.8	46.2
	A01AD11	Various	13	17.1	31.4	68.6
	A11BA	Multivitamins, plain	6	7.9	46.5	53.5
	A01AD02	Benzydamine	5	6.6	50.5	49.5
	A07AX03	Nifuroxazide	5	6.6	53	47
		Others	14	18.4	41.3	58.7
	**Class Total**		**76**	**100**	**45.8**	**54.2**
B-Blood and Blood Forming		Others	2	100	40.2	59.8
	**Class Total**		**2**	**100**	**40.2**	**59.8**
J-Anti-infective (Systemic)	J01CR02	Amoxicillin+Beta-Lactamase Inhibitor	35	64.8	54.3	45.7
	J01FA09	Clarithromycin	9	16.7	61.3	38.7
		Others	10	18.5	54.8	45.2
	**Class Total**		**54**	**100**	**55.6**	**44.4**
M-Musculoskeletal System	M01AE01	Ibuprofen	73	88	49.7	50.3
	M01AE51	Ibuprofen, combinations	10	12	64.2	35.8
	**Class Total**		**83**	100	**51.5**	**48.5**
N-Nervous System	N02BE01	Paracetamol	77	96.3	46.1	53.9
		Others	3	3.7	50.5	49.5
	**Class Total**		**80**	**100**	**46.3**	**53.7**
P-Antiparasitic, I.R.	P01AB01	Metronidazole	11	100	46.1	53.9
	**Class Total**		**11**	**100**	**45.1**	**54.9**
R-Respiratory System	R05X	Other Cold Preparations	85	34	47.6	52.4
	R06AX17	Ketotifen	34	13.6	48.3	51.7
	R05DB13	Butamirate	25	10	40	60
	R05DB27	Levodropropizine	24	9.6	54	46
	R01AA07	Xylometazoline	16	6.4	66.2	33.8
	R06AE07	Cetirizine	12	4.8	19.5	80.5
	R06AX27	Desloratadine	11	4.4	40.8	59.2
	R05CB03	Carbocisteine	5	2	23.8	76.2
	R05DB07	Oxolamine	5	2	49.2	50.8
	R06AB05	Pheniramine	5	2	35.4	64.6
		Others	28	11,2	45.2	54.8
	**Class Total**		**250**	**100**	**46.1**	**53.9**
S- Sensory Organs		Others	2	100	60	40
	**Class Total**		2	100	60	40
**Total**			**558**	**100**	**48.3**	**51.7**

Used and Unused: Percentage (%). Medicines with less than 5 drugs are given as “others”. **A-Others (n = 14)**: A03FA03-Domperidone 4, A03AX13-Silicones 3, A07AA02-Nistatin, A02BX13-Alginic Acid 2, A03AA05-Trimebutine 1, A11CC05-Colecalciferol 1. **B-Others (n = 2)**: B03AE03- Iron and Multivitamins 1, B03AE10-Various Combinations 1. **J-Others (n = 10)**: J05AH02-Oseltamivir 4, J01CR04-Sultamicillin 2, J01DD08-Cefixime 1, J01DD15-Cefdinir 1, J01CE10-Benzathine Phenoxymethylpenicillin 1, J01FA10 Azithromycin 1. **N-Others (n = 3)**: N02BB02-Metamizole sodium 2, N05BB01-Hydroxyzine 1. **R-Others (n = 28)**: R01AD09-Mometasone 4, R01BA02-Pseudoephedrine 4, R03CC02-Salbutamol 4, R05CB01-Acetylcysteine 4, R01AD11-Triamcinolone 3, R03CC53-Terbutaline 3, R05DB20-Combinations 1, R01AD08-Fluticasone 1, 02AX01-Flurbiprofen 1, R05CB15-Erdosteine 1. **S-Others (n = 2)**: S01GX06-Emedastine, S02CA06- Dexamethasone and anti-infectives 1.

It was observed that R-Respiratory System group had the highest number of unused drugs (n = 250 and 44.8%), followed by M-Musculoskeletal System (n = 83 and 14.9%) and N-Nervous System (n = 80 and 14.3%). R05X-Other Cold Preparations (n = 85 and 15.2%), N02BE01-Paracetamol (n = 77 and 13.8%) and M01AE01-Ibuprofen (n = 73 and 13.1%) had the highest number of active ingredients (respectively). The highest percentage of unused drugs were R06AE07-Cetirizine (80.5%), R05CB03-Carbocisteine (76.2%) and A01AD11-Various (68.6%). The percentage of unused drugs in the J-Anti-infective System class was 44.4% and the most common drug in this class was J01CR02-Amoxicillin and Beta-Lactamase Inhibitor (n = 35). According to ATC, the percentage of unused medicines by volume for all drugs was 51.7% (**Table 4).**

## Discussion

In this study, parental practices and perspectives regarding unused pediatric pharmaceuticals, along with the amount and classification of unused drugs, were discussed, and the prominent findings were highlighted.

### Sociodemographic characteristics

On average, the number of unused drugs was higher in male, district, low income, secondary education, non-working and parents with male children. Statistical difference was found in the parameters of place of residence, education level and employment status. Studies have reported that there are some determinants in giving medicines at home to the child. These determinants are parameters such as working status, place of residence, socio-economic status, educational status, etc. [26–28]. It is possible to say that similar characteristics occur in our study and support the existing literature in terms of participants in Türkiye. Therefore, we believe that the addition of person, place and time characteristics when addressing this and similar studies epidemiologically will be a correct approach in terms of studies addressing this issue and will better describe the researched subject.

### Practices and perspectives on unused pediatric medications

The distribution of the number of unused pediatric pharmaceuticals was associated with location and level of education. It was found that unused pediatric pharmaceuticals were kept in the kitchen and refrigerator where children could reach, most of the medicines were prescribed by family physicians, kept at home due to emergencies and the possibility of re-administration, early recovery was one of the most important reasons for unused medication, the behavior of keeping the medication at home until the expiration date and then throwing it away, and the opinion of environmental damage caused by inappropriate disposal was found to be higher than other options. In addition, the mother was found to be the parent who administered medication to the child the most. When the literature is examined, it is clear that this issue is fundamentally related to individual behaviors. A similarity is observed between our findings regarding parental practices and the findings reported in studies on medication-related behaviors among the general population [6,21,29]. This similarity may be related to similar living spaces, behaviors and practices. The difference in frequencies and percentages may be related to the type, time, participant characteristics, sample size, location and culture of the study.

### Self-medication

Two different prevalence’s of self-medication were calculated in this study. One was assessed as *“participant opinion”* (82.4% and most commonly administered antipyretics, analgesics and antipyretics and cold drugs) and the other was measured by asking the participant for each drug during the medication measurement and summarized as *“participant practice”* (58.4% and most commonly administered decongestants, analgesics and antipyretics and cold drugs). In addition, while the prevalence of self-medication of antibiotics was 1.3% in *“participant opinion”,* it was measured to be 40.3% in “practice”, showing a significant difference between opinion and practice. Studies present the prevalence of self-medication as a general finding, sometimes summarizing data on all drugs and sometimes summarizing a specific topic such as antibiotics, and findings vary from country to country. The prevalence of self-medication was reported to be 68% and 92.9% in Morocco [19,30], 20.6% in Tunisia [31], 77.9% in Rwanda [32], 70% in Romania [33], 14.3% in China [34] and 8–34% in a review [18]. The above-mentioned literature has reported that self-medication is associated with multiple factors. These factors include low socioeconomic status, drug purchasing, distance to health facilities, etc. The finding obtained in practice can be considered as an average finding in the literature, but it is possible to evaluate the finding obtained as participant opinion as high for Türkiye. The calculation of two separate self-medication findings in our study is a very important finding (especially the frequency finding obtained for antibiotics), and this may be due to the fact that the participants interpreted the event differently in terms of opinion, and that they could not fully distinguish or recognize the drugs. Therefore, we think that conducting studies on this subject by bringing the participant and the drug side by side, careful monitoring of participant behaviors by the researchers, and face-to-face data collection will clarify the findings.

### Classification of drugs

In 10 of the 13 classes of medicines, the volume of used and unused medicines (ml) was close to each other (p > 0.05), while the volume of unused medicines was high in all other medicines except antibiotics, bronchodilators and non-opioid antitussives. The most important finding of this study was that a median of 56(27–80;53) ml of medication was unused, which corresponded to 51.7% of all medications, and the most important practical reason was early recovery from disease. According to the mechanism of action, it was found that NSAIDs, analgesics and antipyretics and antibiotics were found to be the most commonly kept unused drugs at home. According to ATC, the most unused drugs were classified as R, M and N. The number of studies researching the volume of unused drugs in the literature is very limited. In a previous study conducted by the authors on the general public, the value presented for the amount of unused drugs was 49.4% [21]. It is possible to say that the value obtained from this study is quite close to the previous measurement but higher and that the finding of the amount of unused drugs in the general public and the pediatric population is similar in Türkiye. Similar findings to the current literature were obtained in the evaluation made according to mechanism of action and ATC classification [35–39]. When these findings are interpreted in general, it is possible to say that individuals reflect their own unused drugs and the amount of use to pediatric pharmaceuticals as well. The fact that the most important reason for medications to remain after treatment is early recovery should be especially addressed in this regard. In a study conducted by Pons et al. (2023), it was shown that parents gave medication for easily understandable and intervenable symptoms such as pain and fever [28]. Therefore, discontinuation of the medication by the parent due to the rapid disappearance of the symptoms may increase the amount of medication remaining.

## Conclusion

There are significant amounts of unused medicines at homes and the amounts of used and unused medicines are close to each other. Self-medication is a common behavior in opinion and practice among parents, and the findings regarding antibiotics are remarkable. The medication handling behaviors and viewpoints reported in adult populations show concerning parallels with the findings of this study regarding pediatric medications. Since medication storage areas are largely accessible to children, precautions should be taken against potential risks. Information on unused pediatric pharmaceuticals should be added to trainings on drug use. Individuals, expectant mothers, pregnant women and mothers should be informed about pediatric pharmaceuticals. An economical and efficient drug take-back system should be established in Türkiye.

### Limits of the study

- This study was conducted in one province and as a single center in Türkiye.
- The findings regarding parental practices and perspectives are based on participant self-reports.
- A significant proportion of the participants were women.
- Some medicines available at home could not be included in the study due to the possibility of their use by the participants.
